# DGKγ Knock-Out Mice Show Impairments in Cerebellar Motor Coordination, LTD, and the Dendritic Development of Purkinje Cells through the Activation of PKCγ

**DOI:** 10.1523/ENEURO.0319-19.2020

**Published:** 2020-03-02

**Authors:** Ryosuke Tsumagari, Sho Kakizawa, Sakiko Kikunaga, Yoshitaka Fujihara, Shuji Ueda, Minoru Yamanoue, Naoaki Saito, Masahito Ikawa, Yasuhito Shirai

**Affiliations:** 1 Department of Applied Chemistry in Bioscience, Graduate school of Agricultural Sciences, Kobe University, Kobe 657-8501, Japan; 2 Department of Biological Chemistry, Graduate School of Pharmaceutical Sciences, Kyoto University, Kyoto 606-8501, Japan; 3 Research Institute for Microbial Disease, Osaka University, Osaka 565-0871, Japan; 4 Laboratory of Molecular Pharmacology, Biosignal Research Center, Kobe University, Kobe 657-8501, Japan

**Keywords:** diacylglycerol kinase, knock-out mouse, long-term depression, motor coordination, protein kinase C, Purkinje cells

## Abstract

Diacylglycerol kinase γ (DGKγ) regulates protein kinase C (PKC) activity by converting DG to phosphatidic acid (PA). DGKγ directly interacts with PKCγ and is phosphorylated by PKCγ, resulting in the upregulation of lipid kinase activity. PKC dysfunction impairs motor coordination, indicating that the regulation of PKC activity is important for motor coordination. DGKγ and PKC are abundantly expressed in cerebellar Purkinje cells. However, the physiological role of DGKγ has not been elucidated. Therefore, we developed DGKγ knock-out (KO) mice and tested their cerebellar motor coordination. In DGKγ KO mice, cerebellar motor coordination and long-term depression (LTD) were impaired, and the dendrites of Purkinje cells from DGKγ KO mice were significantly retracted. Interestingly, treatment with the cPKC inhibitor Gö6976 (Gö) rescued the dendritic retraction of primary cultured Purkinje cells from DGKγ KO mice. In contrast, treatment with the PKC activator 12-*o*-tetradecanoylphorbol 13-acetate (TPA) reduced morphologic alterations in the dendrites of Purkinje cells from wild-type (WT) mice. In addition, we confirmed the upregulation of PKCγ activity in the cerebellum of DGKγ KO mice and rescued impaired LTD in DGKγ KO mice with a PKCγ-specific inhibitor. Furthermore, impairment of motor coordination observed in DGKγ KO mice was rescued in tm1c mice with DGKγ reexpression induced by the FLP-flippase recognition target (FRT) recombination system. These results indicate that DGKγ is involved in cerebellar LTD and the dendritic development of Purkinje cells through the regulation of PKCγ activity, and thus contributes to cerebellar motor coordination.

## Significance Statement

The only output neurons of the cerebellum, Purkinje cells, regulate long-term depression (LTD). LTD and the dendritic development of Purkinje cells are important for motor function. Many studies have revealed functional correlations between signaling molecules involved in the mechanisms of motor coordination; however, we still do not have enough knowledge to understand the overall concept. Here, we show that the functional correlation between diacylglycerol kinase γ (DGKγ) and protein kinase Cγ (PKCγ) contributes to motor coordination. DGKγ regulates the activity of PKCγ which is an important factor in LTD and the dendritic development of Purkinje cells, allowing PKC to function precisely as a mediator of motor coordination.

## Introduction

Diacylglycerol (DG) is produced by the hydrolyzis of phosphatidylinositol-4,5-bisphosphate (PIP_2_) by phospholipase C (PLC) in response to neurotransmitters, growth factors and hormones. DG functions as a lipid messenger and activator, by binding to several enzymes including protein kinase C (PKC; Almena and Mérida, 2011). DG signaling is terminated by DG kinase (DGK), which phosphorylates DG to produce phosphatidic acid (PA; Sakane et al., 2007). In other words, DGK indirectly inhibits PKC activity by reducing the level of DG. PA is also an important lipid messenger that activates various enzymes including phosphatidylinositol-4-phosphate-5-kinase (PI4P5K; Jones et al., 2000), Raf1-kinase (Ghosh et al., 1996), mammalian target of rapamycin (mTOR; Fang et al., 2001), and atypical PKC (Limatola et al., 1994). Therefore, DGK is thought to have important physiological roles in response to different stimuli.

To date, 10 mammalian subtypes of DGK have been identified (Topham and Prescott, 1999; Kanoh et al., 2002; van Blitterswijk and Houssa, 2000). Among them, at least 8 subtypes are expressed in the brain with subtype-specific expression patterns, suggesting subtype-specific functions in neurons (Raben and Tu-Sekine, 2011, Shirai and Saito, 2014). For example, DGKγ is abundantly expressed in cerebellar Purkinje cells (Goto and Kondo, 1999; Adachi et al., 2005). However, its function in neurons is still unclear.

Similarly, PKC has 10 subtypes and many of them show subtype-specific expression in the brain. For example, PKCα and γ are expressed in cerebellar Purkinje cells (Metzger and Kapfhammer, 2003) and regulate Purkinje cell function, including synaptic plasticity and dendritic development (Hirai, 2018). The activation of PKC induces long-term depression (LTD) and single climbing fiber (CF) innervation to Purkinje cells, which are important for motor coordination (Ito, 2002; Watanabe and Kano, 2011). PKCα knock-down blocks LTD (Leitges et al., 2004), and PKCγ deficiency and constitutively active mutant PKCγ induce the impairment of cerebellar motor coordination, including the loss of LTD, abnormal dendritic morphology and multiple CF innervation (Chen et al., 1995; Kano et al., 1995; Shuvaev et al., 2011). These reports indicate that the regulation of PKC activity is important for cerebellar motor coordination.

Interestingly, a recent study previously reported that DGKγ directly interacts with PKCγ and is phosphorylated by PKCγ, resulting in the upregulation of DGKγ activity and the subsequent attenuation of PKC activity (Yamaguchi et al., 2006). These facts suggest that DGKγ also has important roles in cerebellar motor coordination. However, its physiological role in cerebellar motor coordination has not been elucidated. Therefore, we investigated the function of DGKγ in cerebellar motor coordination and its molecular mechanism using DGKγ knock-out (KO) mice. We found that DGKγ contributes to motor coordination, the induction of cerebellar LTD and the dendritic development of Purkinje cells through the regulation of PKCγ.

## Materials and Methods

### Materials

Primers were purchased from Thermo Fisher Scientific. We used the following antibodies: rabbit anti-DGKγ (Adachi et al., 2005), rabbit anti-calbindin, rabbit anti-PKC substrate (Cell Signaling), rabbit anti-phospho-PKCγ T674 (Bios), rabbit anti-phospho-PKCα S657 (Abcam), rabbit anti-PKCγ, mouse anti-PKCα, mouse anti-GAPDH (Santa Cruz), mouse anti-β-actin (BD), peroxidase-conjugated AffiniPure goat anti-rabbit and anti-mouse IgG and Alexa Fluor 546 (Alexa 546)-conjugated goat anti-rabbit IgG (Jackson). We used the following cell culture reagents: Sumitomo Nerve-Cell Culture System (Sumitomo Bakelite) and 3,3′,5′-triiodo-l-thyronine (T3) sodium salt (Thermo Fisher Scientific). The plasmids, 12-*o*-tetradecanoylphorbol 13-acetate (TPA), GF109203X (GFX), and Gö6976 (Gö) were donated by Dr. Saito (Biosignal Research Center, Kobe University, Kobe, Japan). Scutellarin (Scu) was purchased from Namiki Shoji.

### Mice

Wild-type (WT) mice (C57BL/6N) were purchased from Japan SLC, Inc. DGKγ KO (tm1a) mice and *CAG-Flpo* transgenic mice were kindly provided by Dr. Ikawa and Dr. Fujihara. Mice were housed under a 14/10 h light/dark cycle with *ad libitum* food and water. All procedures using mice were performed according to the guidelines of the Institute Animal Care and Use Committee. Male six- to 12-week-old mice were used for the motor coordination tests and the electrophysiological experiments.

### Generation of DGKγ KO (tm1a) and floxed (tm1c) mice

A vector targeting the mouse *Dgkg* gene (PRPGS00045_A_B02) was acquired from the International Knockout Mouse Consortium (IKMC; Skarnes et al., 2011). After linearization by *Asi*SI digestion, the targeting vector was electroporated into EGR-G101 ((*CAG/Acr-Egfp*)C57BL/6NCr x (*CAG/Acr-Egfp*)C57BL/6NCr) embryonic stem (ES) cells (Fujihara et al., 2013), and colonies were screened. To disrupt the *Dgkg* gene, exons 4–5 were replaced with an internal ribosomal entry site (IRES): *lacZ* trapping cassette, a floxed promoter-driven neo cassette, and floxed exons 4–5. A diphtheria-toxin-A-chain (DTA) expression cassette was used for negative selection. After G418 selection, four of the 32 drug-resistant clones underwent a homologous recombination event after PCR analysis. The screening primers were: 5′-CACAACGGGTTCTTCTGTTAGTCC-3′ and 5′-GTCATCTAACACAGGAGACCAGTCATG-3′ for the 5’-arm; and 5′-ATCCGGGGGTACCGCGTCGAG-3′ and 5′-GAAGAGACATGAGAGGCAAGATGC-3′ for the 3’-arm. The mutant ES cell clones were injected into eight-cell stage ICR embryos, and the chimeric blastocysts were transferred into the uterine horns of pseudopregnant ICR females the next day. The obtained chimeric males were mated with WT females for germ-line transmission. Offspring from heterozygous intercrosses were genotyped by PCR.

The DGKγ floxed (tm1c) mice, in which the loxP sites flank exons 4–5, were generated by mating DGKγ KO (tm1a) mice with *CAG-Flpo* transgenic mice using the FLP-flippase recognition target (FRT) recombination system. The transgenic mice express an optimized FLP recombinase (FLPo) under the control of a *CAG* promoter (Yamazaki et al., 2016). The genotyping primers were: 5′-GCAACGTGCTGGTTGTTGTGCTGTCTCATC-3′ and 5′-TCAGATCCGCCTGTTGATGTAGCTG-3′. The transgenic allele was amplified by PCR, resulting in a 1352-bp band.

### Genotyping

Genotyping of DGKγ KO (tm1a) and tm1c mice was conducted by PCR using the following primers: 5′-CAGGTGTCTCTTGTCTGGGCT-3′, 5′-TGGGTATAGGGTAGGAACTTGC-3′, and 5′-CACAACGGGTTCTTCTGTTAGTCC-3′. Bands at 907 bp, 523 and 975 bp are expected from WT, DGKγ KO (tm1a) and tm1c mice, respectively. PCR conditions were as follows, 25-μl volume, one cycle at 94°C for 2 min; 40 cycles at 94°C for 1 min, 62°C for 30 s, and 72°C for 1 min; one cycle at 72°C for 10 min.

### Preparation of proteins from the brain

The cerebellum and cerebrum were obtained from WT, DGKγ KO (tm1a), and tm1c mice. These samples were homogenized in ice-cold homogenate buffer [20 mM Tris-HCl, 1 mM EGTA, 1 mM EDTA, 1 mM MgCl_2_, 1 mM phenylmethylsulfonyl fluoride (PMSF), 20 ng/ml leupeptin, 1× phosphatase inhibitor cocktail solution II (Wako), and 1% Triton X-100; pH 7.4] using a handy sonic sonicator (UR-20, Tomy Seiko Co, Ltd.). After centrifugation, the lysates were obtained.

### Western blotting

Western blotting was performed as described previously (Kano et al., 2014). Briefly, the samples were subjected to 10% SDS-PAGE, followed by blotting onto a poly-vinylidene difluoride membrane (Millipore). Nonspecific binding sites were blocked by incubation with 5% skim milk in 0.01 M PBS containing 0.03% Triton X-100 (PBS-T) for 1 h. The membrane was incubated with the appropriate antibody for 1 h at room temperature. After washing with PBS-T, the membrane was incubated with peroxidase-labeled anti-rabbit or anti-mouse IgG for 30 min. After three rinses with PBS-T, the immunoreactive bands were visualized using ImmunoStar (Wako). The densities of the bands were analyzed by ImageJ. To detect phosphorylated proteins, we used 5% BSA instead of skim milk for blocking and 0.01 M TBS containing 0.03% Tween 20 (TBS-T) instead of PBS-T.

### Immunohistochemistry

For immunohistochemical analysis of mouse brains, mice were perfused with 0.9% NaCl through the left ventricle followed by 4% paraformaldehyde (PFA). The brains were removed and immersed for 48 h in 4% PFA at 4°C. The brains were immersed in a 30% sucrose solution for at least 2 d at 4°C. The brains were sliced with a cryostat (Leica CM1850) to produce 20-μm-thick coronal and parasagittal sections. Immunohistochemistry was performed as described previously (Ueda et al., 2013). The sections were incubated in 0.03% H_2_O_2_ in PBS (–) for 1 h and permeabilized with 0.3% Triton X-100 in PBS (–) for 30 min. Subsequently, the sections were washed with 0.1% Tween 20 in PBS (–) and incubated in 0.1% Tween 20 in PBS (–) supplemented with goat serum as a blocking reagent for 1 h. Next, the sections were incubated with an appropriate antibody for 72 h at 4°C. The bound antibodies were visualized using EnVision + System-HRP (Agilent Technology).

### Rotarod test

The rotarod apparatus (MK-630B single lane rotarod, Muromachi Kikai) consisted of a rod (30 mm in diameter, 90 mm wide) flanked by two large round plates (40 cm in diameter). The speed of rotation was increased from four to 40 rotations per minute over 5 min. We recorded the latency for the mice to fall from the rod. The test was performed three times daily for 2 d.

### Footprint test

The hind paws of the mice were coated with nontoxic ink, and the mice were allowed to walk through a tunnel (30 cm long, 16 cm wide, and 17 cm high). The footprint patterns were analyzed for stride length. The test was performed three times.

### Beam test

Mice were trained to traverse an elevated metallic beam (70 cm long, 10 mm in diameter, 60 cm high). They were placed at one end of the beam and an enclosed escape box was placed at the other end. Each hind paw slip was recorded and counted. The test was performed five times daily for 2 d.

### Electrophysiology

Mice were sacrificed by cervical dislocation under anesthesia with diethyl ether. The cerebellum was excised, and parasagittal cerebellar slices (250 μm thick) were prepared from the vermis (Edwards et al., 1989; Kakizawa et al., 2000, 2012). Whole-cell recordings were obtained from visually identified Purkinje cells under an upright microscope (BX51WI, Olympus) using a 40× water-immersion objective at room temperature (23–25°C). The resistance of the patch pipettes was 2.0–3.5 MΩ when filled with an intracellular solution composed of the following: 130 mM K-gluconate, 10 mM KCl, 10 mM NaCl, 1 mM EGTA, 4 mM ATP-Mg, 0.4 mM GTP-Na, and 10 mM HEPES (pH 7.3; adjusted with KOH). The standard bathing solution was composed of the following: 125 mM NaCl, 2.5 mM KCl, 2 mM CaCl_2_, 1 mM MgSO_4_, 1.25 mM NaH_2_PO_4_, 26 mM NaHCO_3_, and 20 mM glucose, bubbled with 95% O_2_ and 5% CO_2_. Bicuculline (10 μM) was always added to block the IPSCs. Scu (100 μM) was added to the extracellular solution for 1 h prior to the stimulation. For focal stimulation of parallel fibers (PFs), a stimulation pipette (with a tip 5–10 μm in diameter) was filled with standard bathing solution and used to apply square pulses (0.1 ms in duration, 0–20 V in amplitude) to the middle portion of the molecular layer. The intensity of each stimulus was adjusted to evoke PF-excitatory postsynaptic currents (EPSCs) with amplitudes of 60–120 pA. The ionic current was recorded from Purkinje cells using a patch-clamp amplifier (EPC-9, HEKA) at a holding potential of −90 or −80 mV, after compensating for liquid junction potential. The signals were filtered at 2 kHz and digitized at 20 kHz.

On-line data acquisition and off-line data analyses were performed using PULSE (HEKA) software. For the LTD experiments, the intensity of the stimulus was adjusted to evoke PF-EPSCs whose initial amplitudes were between 60 and 120 pA. After obtaining a stable initial recording for at least 10 min, conjunctive stimulus (CJS) was applied to induce LTD. The CJS protocol is composed of 300 single PF stimuli in conjunction with depolarizing pulse (−80 to 0 mV for 50 ms) in conjunction with single PF stimuli (at 40 ms after the onset of the pulse) at 1 Hz. The test stimulus was applied to PFs every 10 s. The amplitude of PF-EPSCs was averaged every 60 s, and normalized to the mean value observed 10 min before the LTD-inducing stimulus. A 100 ms, −5 mV hyperpolarizing test pulse preceded each PF stimulus to monitor the series resistance and input resistance of Purkinje cells throughout the experiment. Data were discarded if the resistance changed by >10% (Kakizawa et al., 2012). Data were also discarded when the slope of PF-EPSC amplitude averaged every minute during the initial recording for 10 min was larger than 2% or when the amplitude did not become stable within 20 min after the onset of whole-cell configuration.

For the analysis of membrane capacitance of Purkinje cells, hyperpolarizing current pulse (−80 to −90 mV, for 500 ms) was applied, and the resulting capacitive current was recorded. Membrane capacitances were then calculated according to the model described previously (Llano et al., 1991), which distinguishes two regions of Purkinje cells: region 1 representing the soma and the main proximal dendrites, and region 2 representing the dendritic tree. C1 represents the membrane capacitance of region 1, and C2 represent the capacitance of the dendritic tree of Purkinje cells.

To examine number of CFs innervating the recorded Purkinje cell, we systematically moved the stimulation pipette in the granule cell layer and tried stimulation at 10 different sites. The stimulus intensity was gradually increased at each stimulation site. When a CF was stimulated, an EPSC was elicited in an all-or-none fashion. In some Purkinje cells, more than one discrete CF-EPSC could be elicited when the stimulus intensity was increased or when the stimulating electrode was moved to a different site. The number of CFs innervating the Purkinje cell was estimated by counting the number of discrete CF-EPSC steps elicited in that cell (Kano et al., 1995; Kakizawa et al., 2000, 2003).

### Primary culture of mouse Purkinje cells

Dissociated cerebellar cultures were prepared as described previously (Seki et al., 2009). EXVIII embryos were removed from pregnant WT and DGKγ KO mice anesthetized with Ravonal. The cerebellum was dissected and kept in ice-cold HBSS (Wako). Cerebellar cells were dissociated by using dissociation solutions from the Sumitomo Nerve-Cell Culture System according to the manufacturer’s instructions. Dissociated cerebellar cells were suspended in neuronal culture medium from the Sumitomo Nerve-Cell Culture System, and plated at a concentration of 1 × 10^5^ cells/50 μl in the center of a multi-well glass bottom dish (Matsunami Glass) coated with polyethylenimine (Sigma-Aldrich), and cultured in a humidified atmosphere containing 5% CO_2_ at 37°C. After the cells attached to the bottom (1–2 h later), 250 μl of culture medium was added to each well. The culture medium was supplemented with 100 pm T3. The cells were cultured for 21 d *in vitro* (DIV). Half of the medium was changed every 3–4 d. TPA (200 nM), GFX (200 nM), and Gö (1 μM) were added to the medium for 3 d.

### Confocal microscopy

For immunofluorescent staining, primary cultured Purkinje cells at DIV21 were fixed with 4% PFA for 1 h at 4°C. After fixation, the cells were permeabilized with 0.3% Triton X-100 for 30 min and then blocked with 10% normal goat serum (NGS) for 2 h. The cells were incubated with anti-calbindin antibody for 16 h at 4°C and then visualized with Alexa 546-labeled goat anti-rabbit IgG, followed by observation under confocal microscopy.

The fluorescence of Alexa 546 was observed with a confocal laser scanning fluorescence microscope (FV-500 IX81 Olympus). The images were recorded as TIFF files. To count the number of neurites and branches and their lengths, the images were analyzed with Neurolucida and Neurolucida Explorer software (MBF Bioscience).

### Measurement of PA in the crude synaptosomal membrane fraction

Crude synaptosomes were prepared as described previously (Gu et al., 2009). Briefly, the cerebellum was homogenized in sucrose buffer: 320 mM sucrose, 20 mM Tris-HCl, 1 mM EGTA, 1 mM EDTA, 1 mM MgCl_2_, 1 mM PMSF, 20 ng/ml leupeptin, 1 mM NaF, 1 mM Na_3_VO_4_ and 1× phosphatase inhibitor cocktail solution II; pH 7.4) with a Potter–Elvehjem homogenizer. After centrifugation at 800 × *g* for 5 min, the supernatant was centrifuged at 10,000 × *g* for 10 min. The pellet (crude synaptosome fraction) was homogenized in sucrose buffer containing 1% Triton X-100 and 300 mM NaCl and centrifuged at 16,000 × *g* for 30 min. The supernatant (S2) and pellet (P2) were obtained, and the P2 fraction was dissolved in 1% SDS.

PA was measured as described previously (Morita et al., 2009). Equal amounts of crude synaptosomal membrane fraction (P2) and 1 M NaCl were mixed, and 20 μl of 1% perchloric acid (PCA), 450 μl of chloroform/methanol (1 : 2), 150 μl of chloroform and 150 μl of 1% PCA were added to the samples in order. After centrifugation at 10,000 rpm for 2 min, the lower layer was harvested and dried. The extracts were dissolved in 70 μl of 1% Triton X-100. Then, 30 μl of the solution and 120 μl of reagent buffer A1 (50 mM Tris-HCl, 50 mM NaCl, and 10 kU/ml LPL; pH 7.4) were mixed and reacted for 1 h at 37°C. After incubation for 3 min at 96°C, the mixture was centrifuged at 10,000 rpm for 5 min at room temperature. A total of 50 μl of the supernatant and 50 μl of reagent buffer A2 (50 mM Tris-HCl, 50 mM NaCl, 0.2% Triton X-100, 5 U/ml HRP, and 0.3 mM Amplex Red) were mixed and reacted for 30 min at room temperature. Then, the amount of PA was measured using a Wallac 1420 ARVOsx (PerkinElmer).

### Evaluation of the inhibitory effect of scutellarin on PKCγ and PKCα activities

COS-7 cells were cultured in DMEM supplemented with 10% FBS, penicillin (100 units/ml) and streptomycin (100 μg/ml). The cells were cultured at 37°C in a humidified atmosphere containing 5% CO_2_. A plasmid (PKCγ-GFP or PKCα-GFP) was electroporated into COS-7 cells using a gene pulser (Bio-Rad). After being cultured for 2 d, the cells were preincubated with scutellarin (Scu) for 30 min and then treated with 100 nm TPA for 30 min in Ringer’s solution (5 mM HEPES, 165 mM NaCl, 5 mM KCl, 1 mM CaCl_2_, 1 mM MgCl_2_, and 10 mM glucose; pH 7.4) . After that, the cells were harvested and homogenized in lysis buffer (20 mM Tris-HCl, 1 mM EGTA, 1 mM EDTA, 1 mM MgCl_2_, 1 mM PMSF, 20 ng/ml leupeptin, 5 mM NaF, 2 mM Na_3_VO_4_ 1 mM C_4_H_4_Na_2_O_6_·2H_2_O, and 1 mM C_3_H_7_Na_2_O_6_P·nH_2_O; pH 7.4) using a handy sonic sonicator. After centrifugation, the lysates were subjected to Western blotting using anti-phospho-PKCγ (T674), anti-phospho-PKCα (S657), anti-PKCγ, and anti-PKCα antibodies.

Parasagittal cerebellar slices (300 μm thick) were prepared from the vermis (Edwards et al., 1989; Kakizawa et al., 2000, 2012). The slices were incubated in standard artificial CSF (ACSF; 125 mM NaCl, 2.5 mM KCl, 2 mM CaCl_2_, 1.3 mM MgCl_2_, 1.25 mM NaH_2_PO_4_, 26 mM NaHCO_3_, and 20 mM glucose) for 30 min at 37°C and then incubated for 1 h at room temperature in standard ACSF with or without Scu. The slices were subsequently homogenized in lysis buffer (50 mM Tris-HCl, 150 mM NaCl, 1 mM EDTA, 1 mM PMSF, 20 ng/ml leupeptin, 100 mM NaF, 2 mM Na_3_VO_4_, 20 mM Na_4_P_2_O_7_, 1% Triton X-100, and 10% glycerol; pH 7.5) using a handy sonic sonicator. After centrifugation, the lysates were subjected to Western blotting using anti-phospho-PKCγ (T674), anti-phospho-PKCα (S657), anti-PKCγ, and anti-PKCα antibodies.

### Experimental design and statistical analysis

All animal data were analyzed for male mice. All data are shown as the mean ± SEM, and Student’s *t* test, Dunnett’s test, and Tukey’s multiple comparisons test were used as appropriate to test statistical significance. Data were analyzed using Excel (Microsoft) and R version 3.5.1 (The R Foundation for Statistical Computing). The statistical analysis of multiple CFs was performed using the Kolmogorov–Smirnov test (Igor Pro, Hulkins). Differences were considered significant when *p* < 0.05.

## Results

### Generation of DGKγ KO mice

**Figure 1.**
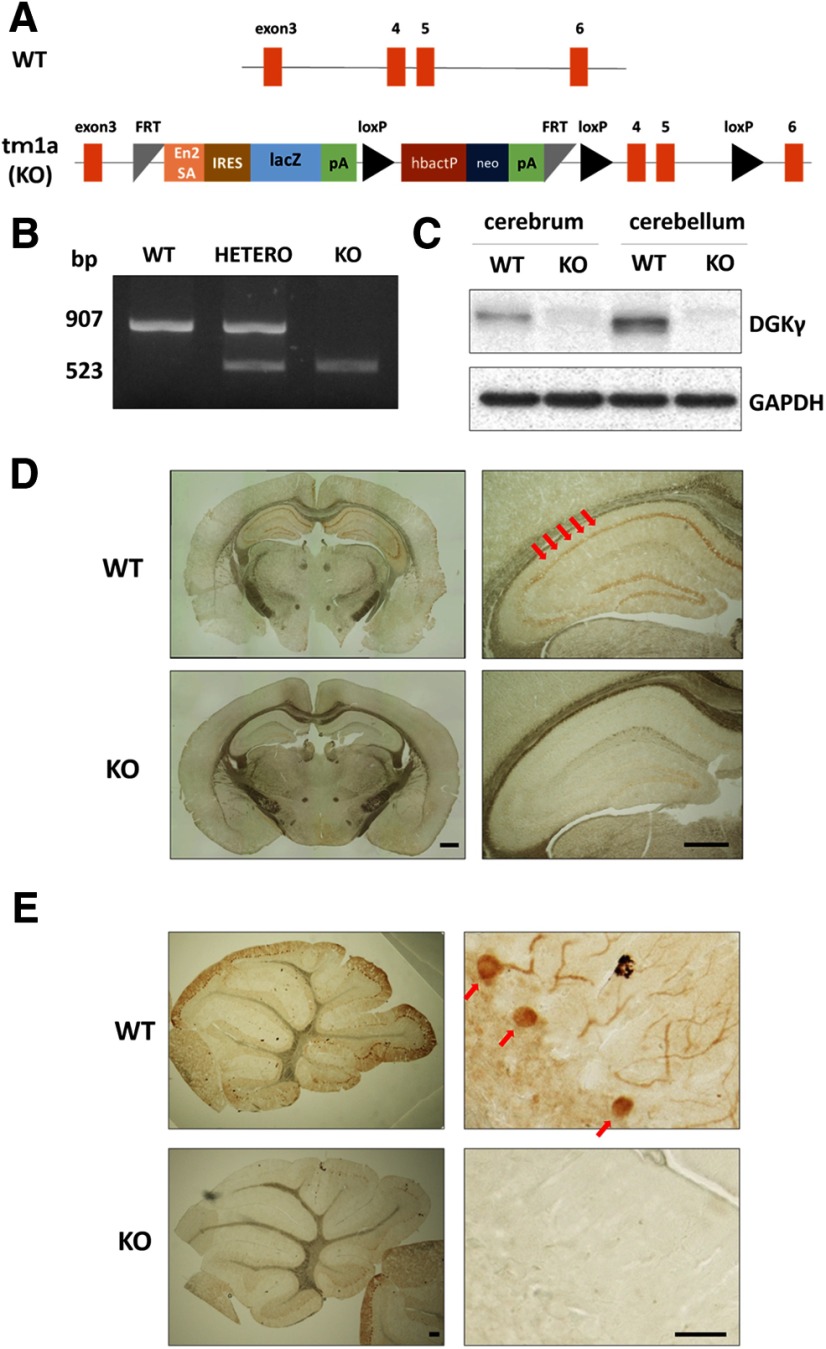
Generation of DGKγ KO mice and PCR genotyping. ***A***, Promotor-driven cassette inserted between exons 3 and 4 of the gene that encodes DGKγ. En2SA, engrailed 2 splice acceptor; lacZ, β-galactosidase; pA, adenovirus polyadenylation signal; loxP, Cre recombinase recognition sequence; hbactP, human-β-actin promotor; neo, neomycin. ***B***, Typical result of PCR genotyping. Bands at 907 and 523 bp were expected for the WT and tm1a alleles, respectively. ***C***, Cerebral and cerebellar lysates from WT and DGKγ KO mice were subjected to Western blotting and probed with an anti-DGKγ antibody. ***D***, ***E***, Coronal sections of the cerebrum (***D***) and parasagittal sections of the cerebellum (***E***) were subjected to immunohistochemistry and stained with an anti-DGKγ antibody. The red arrows show the hippocampus and Purkinje cells. Scale bars: 500 μm (***D***, right and left, ***E***, left) and 100 μm (***E***, right).

We produced homozygous tm1a mice (tm1a/tm1a) by mating tm1a heterozygous mice containing a promotor-driven cassette (Fig. 1*A*). The genotype of the tm1a/tm1a mice was confirmed by PCR (Fig. 1*C*). However, we could not confirm that DGKγ was not expressed in the brains of tm1a/tm1a mice, because the insertion occurred between exons 3 and four of the gene that encodes DGKγ. Therefore, we examined DGKγ expression in the brain by Western blotting and immunohistochemistry using an anti-DGKγ antibody. Western blotting revealed that there was no expression of DGKγ in the cerebrum or cerebellum of tm1a/tm1a mice (Fig. 1*C*). DGKγ immunoreactivity was clearly observed in the hippocampus and cerebellar Purkinje cells and weakly and diffusely expressed in the granule cells of WT mice, but not in those of tm1a/tm1a mice (Fig. 1*D*,*E*). These results indicate that the DGKγ protein was not expressed in the brains of tm1a/tm1a mice; thus, we concluded that these mice were DGKγ KO mice.

### Motor coordination of DGKγ KO mice

**Figure 2.**
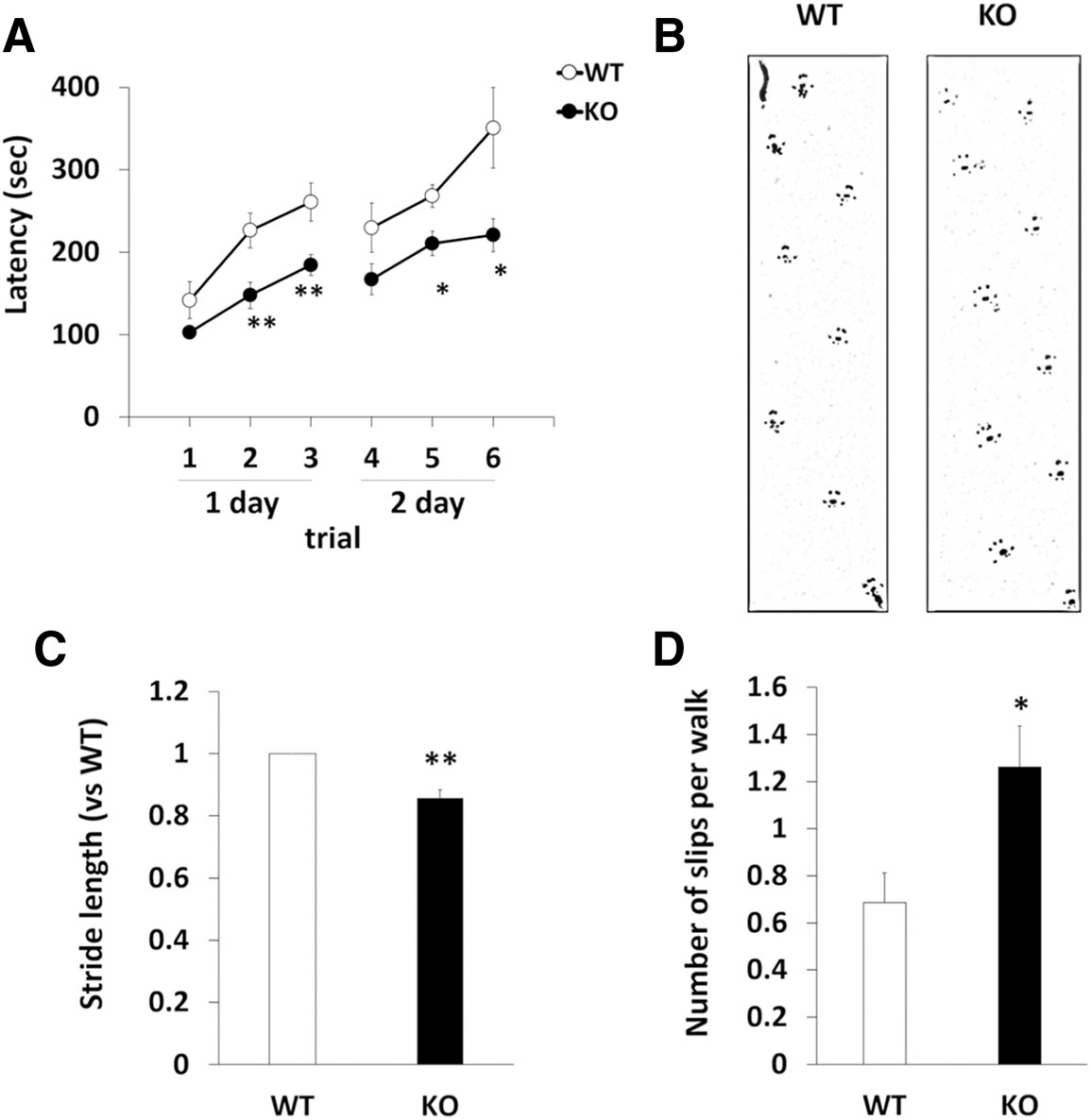
Analysis of motor coordination. ***A***, Motor coordination of WT and DGKγ KO mice was assessed by the accelerating rotarod test. The test was performed three times daily for 2 d (WT: *n* = 9; KO: *n* = 9); *p < 0.05, **p < 0.01, followed by Student’s *t* test. ***B***, ***C***, WT and DGKγ KO mice were allowed to walk through a tunnel, and their footprints were recorded on paper. The ratio of the stride length of DGKγ KO mice to the stride length of WT mice was analyzed (WT: *n* = 5; KO: *n* = 5); **p < 0.01, followed by Student’s *t* test. ***D***, Motor coordination of WT and DGKγ KO mice was assessed by the number of hind paw slips in the beam test. The test was performed five times daily for 2 d (WT: *n* = 7; KO: *n* = 8); *p < 0.05, followed by Student’s *t* test. Data are expressed as mean ± SEM.

DGKγ is abundantly expressed in cerebellar Purkinje cells which are important for motor coordination (Goto and Kondo, 1999; Adachi et al., 2005). Thus, we tested the motor coordination of DGKγ KO mice by the rotarod, footprint and beam tests. In the rotarod test, WT and DGKγ KO mice showed steady improvements over trials, but the latency for DGKγ KO mice to fall from the rod was significantly shorter than that of WT mice (Fig. 2*A*; *p < 0.05, **p < 0.01; Student’s *t* test). In the footprint test, WT and DGKγ KO mice seemed to walk normally (Fig. 2*B*). However, a precise analysis of the hind footprint pattern revealed that the stride length of DGKγ KO mice was significantly (∼15%) shorter than that of WT mice (Fig. 2*C*; *p* = 0.023; Student’s *t* test). In the beam test, DGKγ KO mice exhibited significantly more frequent slips than WT mice (Fig. 2*D*; *p* = 0.033, Student’s *t* test). These results clearly indicate that DGKγ KO mice show impairments in motor coordination.

### LTD in DGKγ KO mice

**Figure 3.**
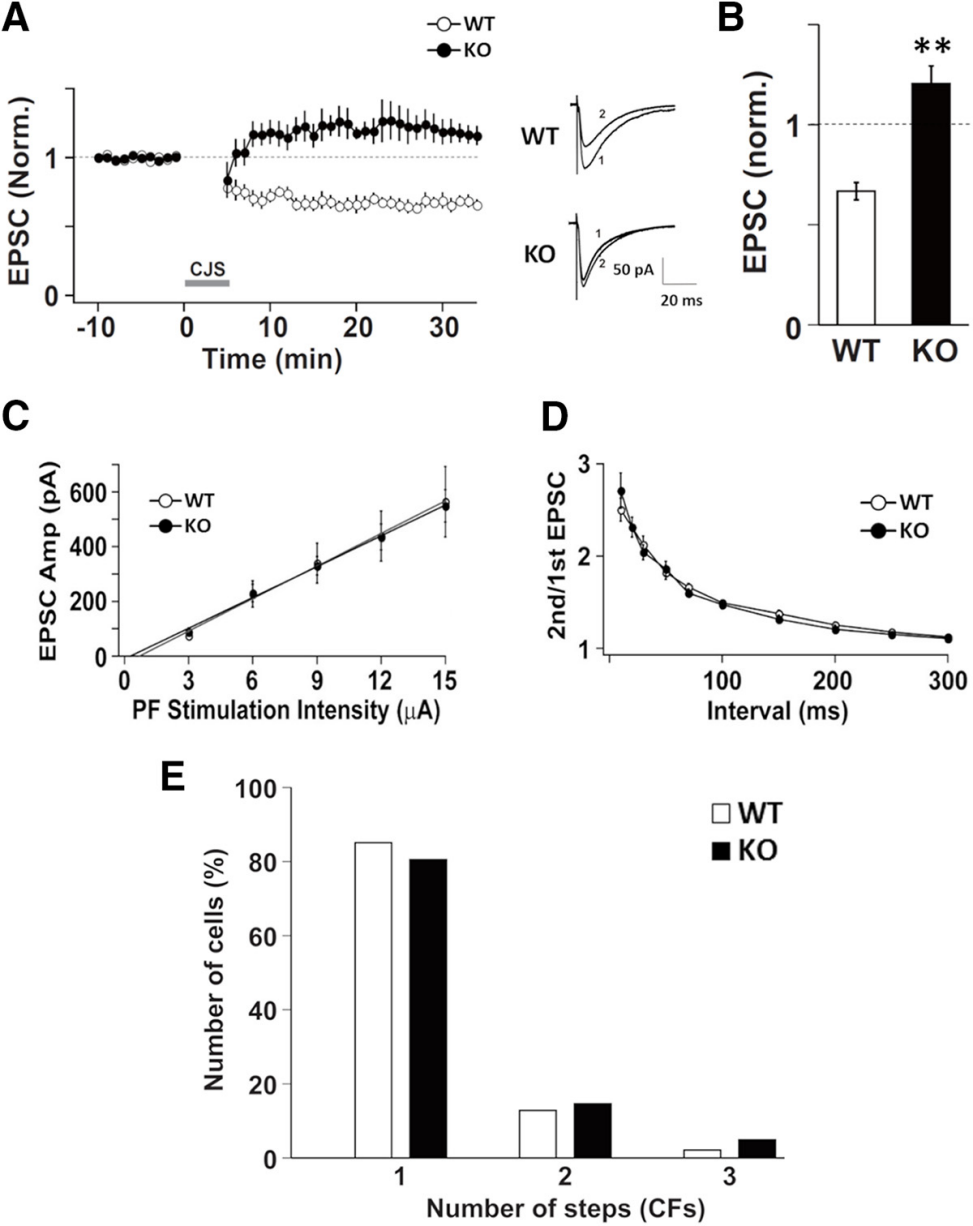
Electrophysiological properties of Purkinje cells in DGKγ KO mice. ***A***, Changes in the PF-EPSC amplitude before and after the CJS of PFs (1 Hz, 300 s) with depolarization applied at time 0. The PF-EPSC amplitude was normalized to the mean over 10 min before CJS (WT: *n* = 5; KO: *n* = 5). Sample traces immediately before (1) and 30 min after (2) CJS. ***B***, Average PF-EPSC amplitude over the 21- to 30-min period after CJS; **p < 0.01, followed by Student’s *t* test. ***C***, The input-output relationship of PF-EPSCs. The PF-EPSCs amplitudes of Purkinje cells from WT and DGKγ KO mice plotted as a function of stimulus intensity (WT: *n* = 11; KO: *n* = 10). ***D***, The PPR of PF-EPSCs recorded from Purkinje cells from WT or DGKγ KO mice at several interpulse intervals (WT: *n* = 11; KO: *n* = 10). ***E***, Summary histograms showing the percentage of discrete steps of CF-EPSCs (WT: *n* = 47; KO: *n* = 41). Data are expressed as mean ± SEM.

Since cerebellar LTD is a basis of motor coordination, we examined cerebellar LTD in DGKγ KO mice. LTD was induced by CJS, which is paired depolarization and parallel fiber (PF) stimulation at 1 Hz for 300 s (Kakizawa et al., 2012), and the amplitude of EPSCs at PF-to-Purkinje cell synapses (PF synapses) after CJS was decreased in WT mice. In contrast, DGKγ KO mice showed impaired LTD (Fig. 3*A*,*B*; *p* = 0.0003, Student’s *t* test); the average EPSC amplitude of DGKγ KO mice 21–30 min after CJS were equal to baseline levels. On the other hand, no significant abnormalities were observed in the electrophysiological properties of PF synapses; there was no significant difference in the input-output relationship, that is, the slope of the EPSC amplitude (output)–stimulus intensity (input) curve, between WT and DGKγ KO mice (Fig. 3*C*; *p* > 0.05, Student’s *t* test). Additionally no significant difference was observed in the paired-pulse ratio (PPR; Fig. 3*D*; *p* > 0.05, Student’s *t* test), an index of the change in transmitter release probability at presynaptic terminals (Zucker and Regehr, 2002). Taken together, the data indicate that, although the basic electrophysiological properties of PF synapses were normal, cerebellar LTD was impaired in DGKγ KO mice.

### CF innervation to Purkinje cells in DGKγ KO mice

CF innervation of Purkinje cells is also responsible for motor coordination. Single Purkinje cells are originally innervated by multiple CFs; however, they are innervated by a single CF as they grow due to pruning (Watanabe and Kano, 2011). A deficiency in the mGluR1-PKCγ signaling cascade results in multiple CF innervation in mature Purkinje cells and subsequent impaired motor coordination (Kano et al., 1995, 1997, 1998; Offermanns et al., 1997). Therefore, we investigated the number of CFs innervating Purkinje cells in DGKγ KO mice. Multiple CF innervation was not observed in DGKγ KO mice (Fig. 3*E*; *p* = 0.473, Kolmogorov–Smirnov test). These results indicate that DGKγ deficiency did not affect CF innervation in Purkinje cells.

### Dendritic development of Purkinje cells in DGKγ KO mice

**Figure 4.**
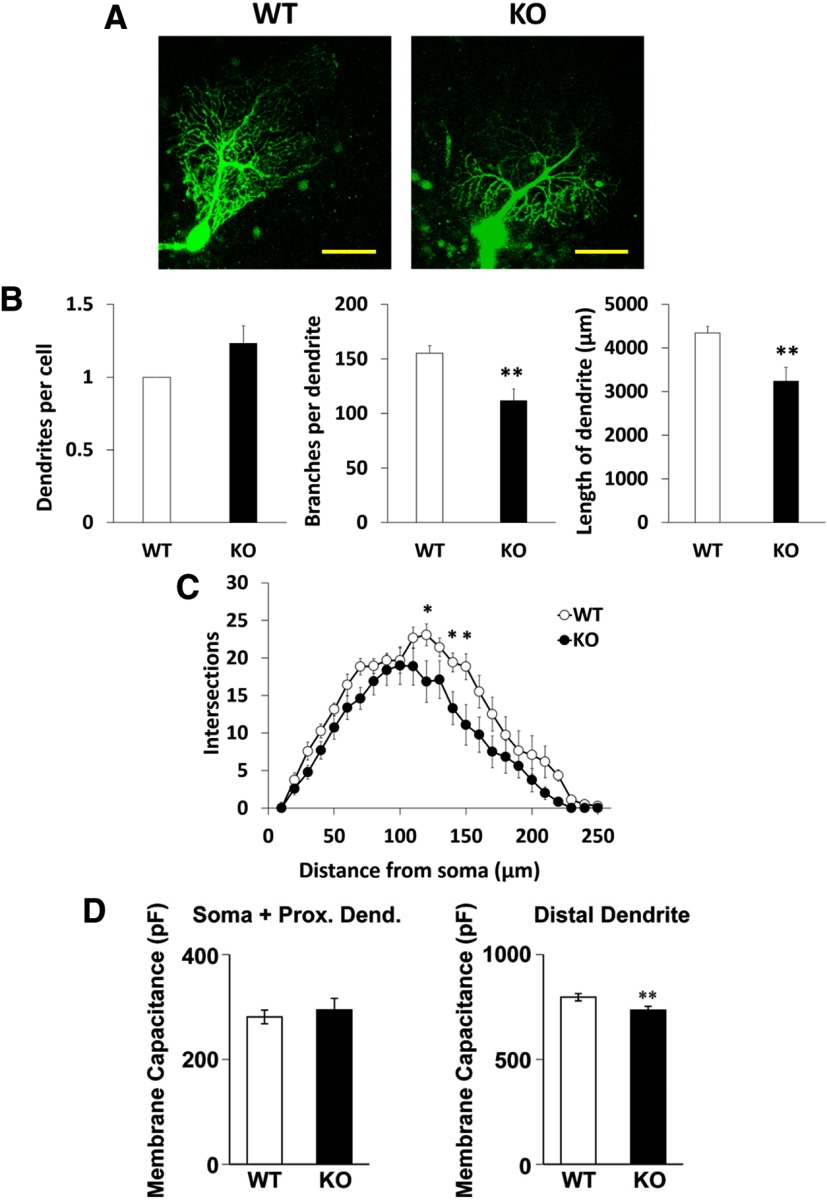
Morphology of Purkinje cells in the cerebellum of DGKγ KO mice. ***A***, Images of Purkinje cells in cerebellar slices from WT and DGKγ KO mice. Purkinje cells were filled with fluorescent dye from the patch pipette. Scale bar: 50 μm. ***B***, A comparison of the number of dendrites and branches and the total length of Purkinje cells from WT and DGKγ KO mice (WT: *n* = 12; KO: *n* = 13); *p < 0.05, **p < 0.01, followed by Student’s *t* test. ***C***, Sholl analysis of the number of intersections of Purkinje cells from WT and DGKγ KO mice (WT: *n* = 12; KO: *n* = 13); *p < 0.05, followed by Student’s *t* test. ***D***, Membrane capacitance of the somatic and proximal dendritic regions (soma + prox. dend.) and the distal dendritic region (distal dendrite) of cerebellar Purkinje cells (WT: *n* = 24; KO: *n* = 21); *p < 0.05, followed by Student’s *t* test. Data are expressed as mean ± SEM.

Next, we investigated the dendritic morphology of Purkinje cells in DGKγ KO mice, because the dendritic morphology of Purkinje cells is also important for motor coordination. Purkinje cells were visualized by filling them with fluorescent dye from the a recording pipette, and impairments in the dendritic development of Purkinje cells were revealed in DGKγ KO mice (Fig. 4*A*); the number of branches and the total length of the dendrites in DGKγ KO mice were significantly lower than those in WT mice, although there was no significant difference in the number of dendrites per cell (Fig. 4*B*). Sholl analysis demonstrated that there were fewer branches in distal dendrites at distances of 120, 140, and 150 μm from the soma in KO mice (Fig. 4*C*; 120 μm: *p* = 0.045, 140 μm: *p* = 0.043, 150 μm: *p* = 0.006, Student’s *t* test).

In some cases, a smaller dendritic region is reflected by decreased membrane capacitance (Kakizawa et al., 2003). Therefore, we subsequently examined the membrane capacitance of Purkinje cells. Although there was no significant difference in the membrane capacitance of the somata and proximal dendrites, the membrane capacitance of the distal dendritic region was significantly lower in DGKγ KO mice than in WT mice (Fig. 4*D*; soma + prox. dend.: *p* = 0.305, distal dendrite: *p* = 0.009, Student’s *t* test). This result is consistent with the impaired morphology of Purkinje cells in DGKγ KO mice.

**Figure 5.**
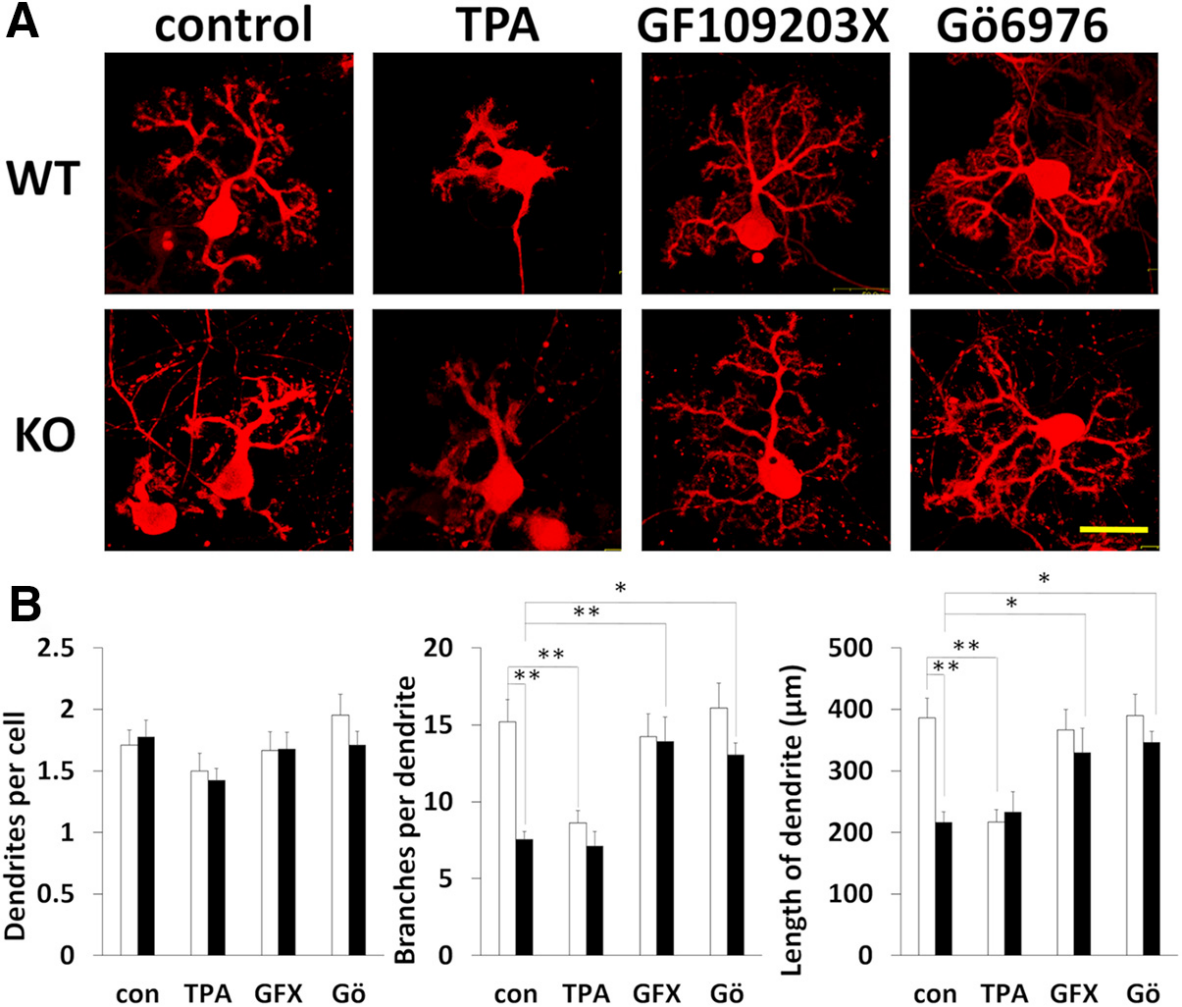
Effects of a PKC inhibitor on the dendritic development of primary cultured Purkinje cells. ***A***, Purkinje cells from WT and DGKγ KO mice were cultured for DIV21. The cells were treated with the PKC activator TPA, the panPKC inhibitor GF109203X (GFX) and the cPKC inhibitor Gö6976 (Gö) for the final 3 d. After fixation, the cells were immunostained with an anti-calbindin antibody. Scale bar: 50 μm. ***B***, A comparison of the number of dendrites and branches and the total length of primary cultured Purkinje cells from WT and DGKγ KO mice (WT, control (con): *n* = 31, TPA: *n* = 22, GFX: *n* = 27, Gö: *n* = 22; KO, con: *n* = 31, TPA: *n* = 26, GFX: *n* = 27, Gö: *n* = 24); *p < 0.05, **p < 0.01, followed by Tukey’s multiple comparisons test. Data are expressed as mean ± SEM.

To confirm dendritic morphologic impairments in Purkinje cells from DGKγ KO mice, we compared morphologic differences between primary cultured Purkinje cells from WT and DGKγ KO mice. Primary cultured Purkinje cells prepared from EXVIII embryos were cultured for 21 DIV and stained with an anti-calbindin antibody (Fig. 5*A*). The number of branches and total length of dendrites of primary cultured Purkinje cells from DGKγ KO mice were significantly decreased compared with those from WT mice (control (con) in Fig. 5*B*, center and right; WT con vs KO con, branches: *p* = 0.000013, length: *p* = 0.000056, Tukey’s multiple comparison test). However, there was no difference in the number of dendrites per cell (con in Fig. 5*B*, left; *p* > 0.05, Tukey’s multiple comparison test). These results confirm the impaired branching of Purkinje cells from DGKγ KO mice.

To investigate the molecular mechanism underlying the morphologic impairment of primary cultured Purkinje cells from DGKγ KO mice, we focused on PKC activity because PKC is involved in the dendritic morphology of Purkinje cells and its activation results in the retraction of dendrites and a reduction in the number of branches (Metzger and Kapfhammer, 2000); an increase in DG due to DGKγ deficiency activates PKC in primary cultured Purkinje cells from DGKγ KO mice. Therefore, we treated primary cultured Purkinje cells with a PKC activator TPA and the panPKC inhibitor GF109203X (GFX) for the final 3 d. These pharmacological treatments did not change the number of dendrites in Purkinje cells from either WT or DGKγ KO mice (Fig. 5*B*, left; *p* > 0.05, Tukey’s multiple comparison test). In contrast, TPA treatment reduced the number of branches and total length of dendrites of Purkinje cells from WT mice but not of Purkinje cells from DGKγ KO mice (TPA in Fig. 5*B*, center and right; WT con vs WT TPA, branches: *p* = 0.0026, length: *p* = 0.0013; KO con vs KO TPA, branches: *p* = 1.00; length: *p* = 0.98, Tukey’s multiple comparison test). On the other hand, the decreased number of branches and total length of dendrites of Purkinje cells from DGKγ KO mice were rescued to WT levels by treatment by the PKC inhibitor GFX (GFX in Fig. 5*B*, center and right; KO con vs KO GFX, branches: *p* = 0.00,041, length: *p* = 0.0066, Tukey’s multiple comparison test). These results indicate that PKC activation is key for the morphologic impairment of primary cultured Purkinje cells from KO mice. To further investigate the PKC subtypes involved in these phenomena, we used the cPKC inhibitor Gö6976. Similar to GFX treatment, Gö treatment rescued the impairments in Purkinje cells from DGKγ KO mice (Gö in Fig. 5*B*, center and right; KO con vs KO Gö, branches: *p* = 0.042, length: *p* = 0.043, Tukey’s multiple comparison test). These results suggest that DGKγ contributes to the dendritic development of Purkinje cells via the regulation of cPKC activity.

### PKCγ activation in the cerebellum of DGKγ KO mice

**Figure 6.**
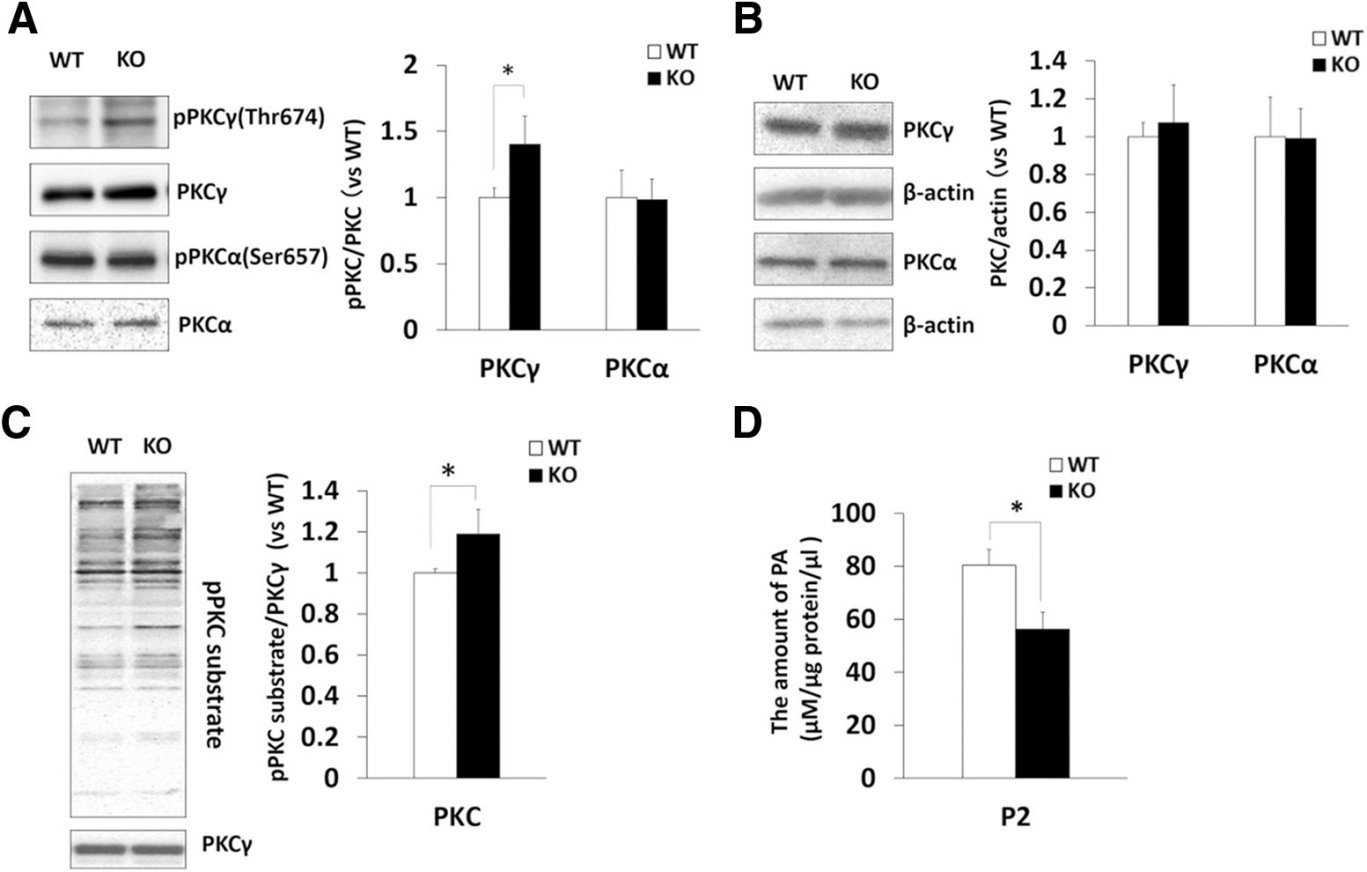
Upregulation of PKC activity in the cerebellum of DGKγ KO mice. ***A*–*C***, Cerebellar lysates from WT and DGKγ KO mice were subjected to Western blotting and probed with anti-PKCγ, anti-PKCα, anti-phospho-PKCγ and anti-phospho-PKCα, anti-β-actin, and anti-PKC substrate antibodies. Quantification of the autophosphorylation and expression levels of PKCγ as well as PKCα and PKC activity was performed by ImageJ. ***A***, Phosphorylation of PKCγ and PKCα was normalized to the PKCγ and PKCα expression levels. ***B***, Expression levels of PKCγ and PKCα were normalized to the expression level of the loading control (β-actin). ***C***, Phosphorylation of the PKC substrate was normalized to the PKCγ expression level. The ratio of the phosphorylation of PKCγ, PKCα, and PKC substrate to the expression levels of PKCγ and PKCα to WT was plotted (PKCγ: *n* = 3; PKCα: *n* = 3; PKC substrate: *n* = 4); *p < 0.05, followed by Student’s *t* test. ***D***, The amount of PA in the P2 (crude synaptosomal membrane) fraction of WT and DGKγ KO mice was measured by an enzymatic method (*n* = 4); *p < 0.05, followed by Student’s *t* test. Data are expressed as mean ± SEM.

Among cPKCs, PKCα and PKCγ are expressed in Purkinje cells and they regulate LTD and the dendritic development of Purkinje cells. To confirm whether the activation of PKC indeed occurs in the cerebellum of DGKγ KO mice *in vivo*, we investigated the autophosphorylation levels of PKCα and PKCγ as hallmarks of PKC activity. The phosphorylation of PKCγ in the cerebellum of DGKγ KO mice was upregulated, while that of PKCα was not changed (Fig. 6*A*; PKCγ: *p* = 0.034 PKCα: *p* = 0.69, Student’s *t* test), indicating that PKCγ was activated in the cerebellum of DGKγ KO mice. The loss of DGKγ did not affect the expression levels of PKCα or PKCγ (Fig. 6*B*; PKCγ: *p* = 0.28; PKCα: *p* = 0.95, Student’s *t* test). Furthermore, we investigated whether PKC is actually activated in the cerebellum of DGKγ KO mice by Western blotting using an anti-PKC substrate antibody that recognizes PKC phosphorylation sites (Fig. 6*C*). Quantification of the band intensity revealed that the phosphorylation of total PKC substrates was elevated in the cerebellum of DGKγ KO mice (Fig. 6*C*; *p* = 0.021, Student’s *t* test), indicating that PKC was indeed activated in DGKγ KO mice.

The upregulation of PKC activity is results from an increase in the DG level induced by DGKγ deficiency. In other words, the PA level is reduced in DGKγ KO mice. Therefore, we measured the level of PA in the P2 (crude synaptosomal membrane) fraction of DGKγ KO mice. The level of PA in the P2 fraction of DGKγ KO mice was significantly decreased compared with that of WT mice (Fig. 6*D*; *p* = 0.033, Student’s *t* test). These results suggest that activated PKCγ causes abnormalities in the dendritic development of Purkinje cells and LTD, resulting in the impairment of motor coordination.

### Effect of a PKCγ inhibitor on impaired LTD in DGKγ KO mice

**Figure 7.**
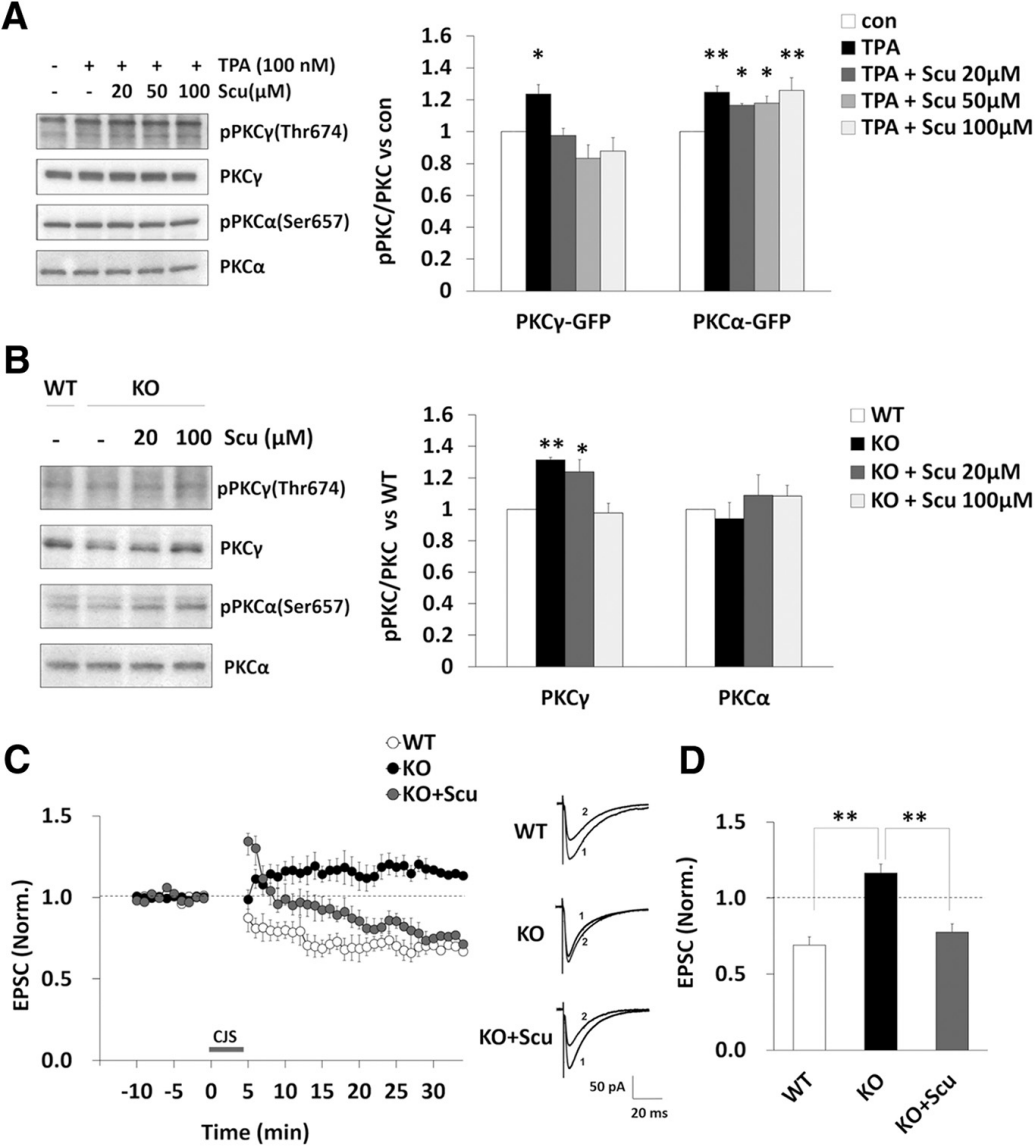
Rescue of impaired LTD in DGKγ KO mice by a PKCγ inhibitor. ***A***, COS-7 cells overexpressing PKCγ-GFP or PKCα-GFP were cultured for 48 h. The cells were treated with TPA (100 nm) for 30 min after preincubation with or without scutellarin (Scu; 20, 50, or 100 μm) for 30 min. The lysates from the cells were subjected to Western blotting and probed with anti-phospho-PKCγ, anti-PKCγ, anti-phospho-PKCα, and anti-PKCα antibodies. Quantification of the autophosphorylation of PKCγ and PKCα was performed by ImageJ. The phosphorylation levels of PKCγ and PKCα were normalized to the PKCγ and PKCα expression levels. The ratio of the phosphorylation of PKCγ and PKCα to that in the control was plotted (PKCγ-GFP: *n* = 6; PKCα-GFP: *n* = 6); *p < 0.05, **p < 0.01 (vs control), followed by Dunnett’s test. ***B***, Acute cerebellar slices from WT and DGKγ KO mice were incubated with or without Scu (20 or 100 μm) for 1 h. Lysates from the slices were subjected to Western blotting as described in ***A*** (PKCγ: *n* = 3; PKCα: *n* = 3); *p < 0.05, **p < 0.01 (vs WT), followed by Dunnett’s test. ***C***, Changes in the PF-EPSC amplitude before and after the CJS of PF (1 Hz, 300 s) with depolarization applied at time 0. A PKCγ inhibitor Scu (100 μm) was added to the extracellular solution prior to stimulation. The PF-EPSC amplitude was normalized to the mean over 10 min before CJS (WT: *n* = 8; KO: *n* = 8; KO+Scu: *n* = 7). Sample traces immediately before (1) and 30 min after (2) CJS. ***D***, Average PF-EPSC amplitude over the 21- to 30-min period after CJS; **p < 0.01, followed by Tukey’s multiple comparisons test. Data are expressed as mean ± SEM.

It is known that PKCα but not PKCγ is important for LTD. However, we found that there was no change in the activation of PKCα and that PKCγ was activated in the cerebellum of DGKγ KO mice in the basal state. To investigate the involvement of abnormal PKCγ activation in impaired LTD in DGKγ KO mice, we used a PKCγ inhibitor. Scutellarin (Scu) is a flavonoid that is a major active ingredient of *Erigeron breviscapus* Hand. Mazz., a plant used in Chinese herbal medicine. A recent study reported that Scu inhibits the translocation of PKCγ from the cytoplasm to the plasma membrane which is a hallmark of PKC activation, but has no effect on the translocation of PKCα (Xu et al., 2007; Su et al., 2012), suggesting that Scu specifically inhibits PKCγ activity but not PKCα activity. Therefore, we tried to confirm its inhibitory effect on the autophosphorylation of PKCγ and PKCα. Stimulating COS-7 cells overexpressing PKCγ-GFP or PKCα-GFP with TPA induced the autophosphorylation of both PKCγ and PKCα (Fig. 7*A*; PKCγ: *p* = 0.047 PKCα: *p* = 0.0021, Dunnett’s test). However, pretreating the cells with Scu significantly inhibited the autophosphorylation of PKCγ but not the autophosphorylation of PKCα in a dose-dependent manner, but not the autophosphorylation of PKCα ((Fig. 7*A*; PKCγ, 20 μM: *p* = 0.99, 50 μM: *p* = 0.21, 100 μM: *p* = 0.47; PKCα, 20 μM: *p* = 0.046, 50 μM: *p* = 0.027, 100 μM: *p* = 0.0012, Dunnett’s test). Then, we also investigated the inhibitory effect of Scu on PKCγ activity in cerebellar slices from DGKγ KO mice. Scu (100 μM) normalized the upregulated PKCγ activity seen in DGKγ KO mice to WT levels, but it had no significant effect on PKCα activity (Fig. 7*B*; PKCγ, KO: *p* = 0.0059, 20 μM: *p* = 0.0025, Dunnett’s test). These results suggest that Scu can be used as a PKCγ inhibitor. Finally, to investigate the effect of a PKCγ inhibitor on impaired LTD in DGKγ KO mice, we added Scu to the extracellular solution when we measured LTD. As expected, Scu rescued the impairment of LTD in DGKγ KO mice (Fig. 7*C*,*D*; WT-KO: *p* = 0.00,002; KO-KO+Scu: *p* = 0.00,017, Tukey’s multiple comparison test). These results indicate that the impaired LTD in DGKγ KO mice is dependent on PKCγ activation in the basal state.

### Rescue effect of DGKγ on the impairment of motor coordination

**Figure 8.**
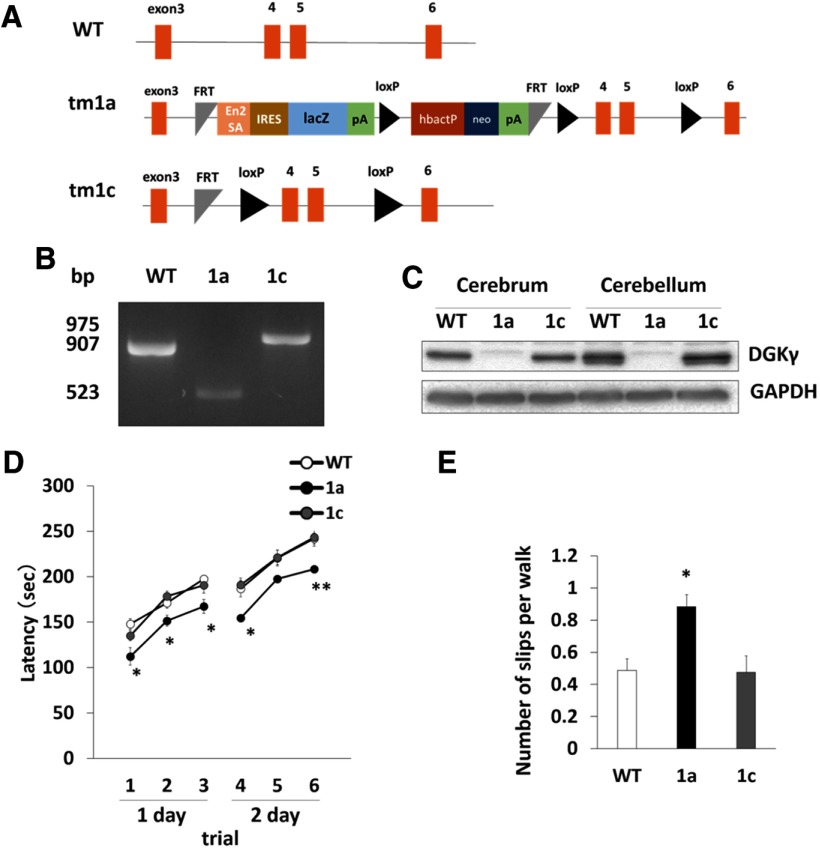
Rescue of motor coordination in DGKγ floxed (tm1c) mice. ***A***, WT, tm1a, and tm1c alleles. ***B***, Typical results of PCR genotyping. Bands at 975, 907, and 523 bp were expected for the tm1c, WT, and tm1a alleles, respectively. ***C***, Cerebral and cerebellar lysates from WT, tm1a (1a), and tm1c (1c) mice were subjected to Western blotting and probed with anti-DGKγ and anti-GAPDH antibodies. ***D***, Motor coordination of WT, tm1a (1a), and tm1c (1c) mice was assessed by the accelerating rotarod test (WT: *n* = 14; 1a: *n* = 15; 1c: *n* = 15); *p < 0.05, **p < 0.01, followed by Tukey’s multiple comparisons test. ***E***, Motor coordination of WT, tm1a (1a), and tm1c (1c) mice was assessed by the number of hind paw slips in the beam test (WT: *n* = 5; 1a: *n* = 5; 1c: *n* = 3); *p < 0.05, followed by Tukey’s multiple comparisons test. Data are expressed as mean ± SEM.

Finally, we performed a rescue experiment using the FLP-FRT recombination system. As shown in Figure 8*A*, the cassette was inserted into DGKγ KO mice (tm1a/tm1a), and we eliminated the cassette by mating DGKγ KO mice with FLP mice. Recombination was confirmed by PCR (Fig. 8*B*), and the recovery of DGKγ in the cerebrum and cerebellum of tm1c/tm1c (1c) mice was confirmed by Western blotting (Fig. 8*C*). The rotarod and beam tests showed that the motor coordination of the 1c mice was normal (Fig. 8*D*,*E*; *p < 0.05, **p < 0.01; Tukey’s multiple comparison test), suggesting the importance of DGKγ for motor coordination.

## Discussion

DGKγ and PKCγ are abundantly expressed in cerebellar Purkinje cells, and the functional correlation between these kinases is important for cerebellar motor coordination and the dendritic development of Purkinje cells. In this study, we investigated the function of DGKγ in cerebellar motor coordination and related molecular mechanism using newly developed DGKγ KO mice. Cerebellar motor coordination was impaired in DGKγ KO mice most likely due to the loss of LTD and the impairment in the dendritic development of Purkinje cells. Our results suggest that DGKγ contributes to cerebellar motor coordination through the regulation of LTD and the dendritic development of Purkinje cells through the regulation of PKCγ activity.

To clarify the mechanism underlying the impairment of LTD in DGKγ KO mice, we investigated the activity of PKCα because it is responsible for inducing LTD through the phosphorylation of GluR2 S880 (Leitges et al., 2004). However, PKCα activation in the cerebellum was not detected. We also checked the phosphorylation of the S880 residue of GluR2, and found that the phosphorylation of GluR2 in the cerebellum of DGKγ KO mice was not changed compared with that in the cerebellum of WT mice (data not shown), confirming that PKCα was not upregulated in the cerebellum in the basal state. On the other hand, PKCγ was activated in the cerebellum of DGKγ KO mice. Why only PKCγ was upregulated when both PKCγ and PKCα can be activated by DG and Ca^2+^. This may have been because PKCγ but not PKCα can be activated by DG at a basal intracellular Ca^2+^ concentration (Kohout et al., 2002; Ananthanarayanan et al., 2003). Another possibility is the mutual regulation of DGKγ and PKCγ; DGKγ and PKCγ directly interact, and the phosphorylation of DGKγ by PKCγ upregulates DGKγ activity (Yamaguchi et al., 2006). In addition, DGKγ accelerates the retranslocation of PKCγ from the membrane to the cytoplasm (Shirai et al., 2000), indicating that DGKγ and PKCγ mutually regulate each other’s activity and that DGKγ negatively regulates PKCγ activity under basal conditions. There have been several reports on the mutual regulation of other DGKs and PKCs (Topham et al., 1998; Imai et al., 2004; Van Baal et al., 2005) and the importance of these regulator mechanisms for synaptic plasticity (Lee et al., 2016). For example, DGKζ directly interacts with PKCα and lowers PKCα activity by reducing DG levels in the basal state (Luo et al., 2003). An increase in the DG levels activates PKCα, which phosphorylates DGKζ, resulting in the dissociation of PKCα and DGKζ. The spatial regulation of PKCα activity by DGKζ contributes to cerebellar LTD (Lee et al., 2015). Therefore, PKCα in the cerebellum of DGKγ KO mice may be inhibited by its interaction with DGKζ. Furthermore, PKCs have different sensitivities and dependencies on DG species (Kamiya et al., 2016), and each DGK utilizes different DG species (Sakane et al., 2018). Taken together, DGKγ deficiency may upregulate PKCγ but not PKCα. However, the upregulation of both PKCα and PKCγ is likely induced by an increase of DG levels through DGKγ deficiency. Indeed, the PKCγ inhibitor Scu rescued impaired cerebellar LTD in DGKγ KO mice. These results suggest that PKCγ upregulation in the basal state is involved in the impairment of LTD in DGKγ KO mice and that DGKγ is responsible for lowering PKCγ activity at LTD stimulation.

In this case, a new question of how upregulated PKCγ impairs cerebellar LTD has arisen. Transient receptor potential canonical channel 3 (TRPC3) may be a key molecule. TRPC3 is abundantly expressed in Purkinje cells, and PKCγ negatively regulates TRPC3 activity and extracellular Ca^2+^ influx (Adachi et al., 2008). TRPC3 KO mice show dysfunction of motor coordination, and the inhibition of TRPC3 impairs cerebellar LTD (Hartmann et al., 2008; Kim, 2013). PKCγ negatively regulates TRPC3 activity and extracellular Ca^2+^ influx through TRPC3 (Adachi et al., 2008). Based on these facts, we speculate that the inactivation of TRPC3 by upregulated PKCγ may inhibit Ca^2+^ influx, resulting in PKCα inactivation during LTD stimulation. LTD stimulation increases the DG levels and the intracellular Ca^2+^ concentration through the mGluR1 cascade and depolarization (Ito, 2002); however, the latter but not the former controls PKCα activation during the induction of cerebellar LTD (Tsuruno and Hirano, 2007). In other words, the Ca^2+^ influx into Purkinje cells in DGKγ KO mice is not enough to activate PKCα and release PKCα and DGKζ. This speculation does not deny the involvement of PKCα in cerebellar LTD. However, to confirm this hypothesis, additional experiments are needed.

Our results also showed that impairments in the dendritic development of Purkinje cells in DGKγ KO mice and the cPKC inhibitor Gö6976 normalized the dendrite retraction of Purkinje cells in DGKγ KO mice to WT levels, suggesting that cPKC activity mainly functions in the dendritic development in Purkinje cells. In particular, the activity of PKCγ is likely involved in the impairment of Purkinje cell branching in DGKγ KO mice because the activation of PKCγ was confirmed. Indeed, the dendrites of Purkinje cells in PKCγ KO mice are enlarged, whereas those in PKCα KO mice are not changed compared with those in WT mice (Schrenk et al., 2002; Gundlfinger et al., 2003). Furthermore, DGKγ activity was necessary for the development of neurites in Neuro-2A cells (data not shown). Taken together, these results suggest that DGKγ contributes to the dendritic development of Purkinje cells through the regulation of PKCγ activity. In addition, PKCγ regulates spinogenesis and the morphology of the spines of distal dendrites through the phosphorylation of CaMKIIβ (Sugawara et al., 2017). Purkinje cells in DGKγ KO mice showed retracted distal dendrites, but the basic electrophysiological properties of PFs were normal, indicating that DGKγ KO mice retained basic excitatory circuitry. On the other hand, morphologic abnormalities in spines of Purkinje cells cause impairments in motor coordination (Sugawara et al., 2013). Accordingly, morphologic abnormalities in spines of Purkinje cells may also contribute to cerebellar motor dysfunction in DGKγ KO mice, although further study is necessary.

Cerebellar motor coordination was impaired not only by cerebellar LTD and alterations in the dendritic morphology of Purkinje cells but also by the disruption of refined motor program transmission through multiple CF innervation. A single Purkinje cell originally exhibits multiple CF innervation; however, it exhibits mono CF innervation as it grows due to pruning (Watanabe and Kano, 2011). CFs transmit signals to Purkinje cells to refine the motor program to prevent incorrect motor programming, resulting in the suppression of PF-EPSCs involved in incorrect motor programming and the induction of cerebellar LTD (Welsh et al., 1995). Previous reports have indicated the importance of PKCγ in CF innervation; multiple CF innervation has been detected in mature PKCγ KO mice (Kano et al., 1995), and a constitutively active PKCγ mutant (S119P) also causes multiple CF innervation (Shuvaev et al., 2011). However, we demonstrated that the CF innervation of Purkinje cells in DGKγ KO mice was normal, although PKCγ activation was detected in the cerebellum. This is not surprising because the loss of function of PKCγ seems to be critical for CF innervation. Indeed, the membrane residence time of the PKCγ mutant (S119P) is shorter due to a lack of binding with DG induced by the mutation in the C1 domain; that is, the PKCγ mutant does not exhibit activity in the membrane (Adachi et al., 2008). Alternatively, PKCγ activity in the CFs of DGKγ KO mice may be normal because CFs do not express DGKγ, even in WT mice.

Thus far, we have focused on PKC to understand the molecular mechanism underlying the impairment of cerebellar LTD and dendritic development of Purkinje cells in DGKγ KO mice; these processes are activated by DG but we also studied PA. Our results showed that PA was decreased in the cerebellum of DGKγ KO mice, suggesting that aberrant PA-mTOR signaling has some influence on impaired motor coordination in DGKγ KO mice. PA, which is generated by DGK, activates mTOR (Avila-Flores et al., 2005), which is involved in neurodevelopment and neuropsychiatric disorders (Costa-Mattioli and Monteggia, 2013; Bockaert and Marin, 2015). mTOR forms two distinct complexes, namely, mTORC1 and mTORC2, which are distinguished by their specific components and sensitivity to rapamycin (Laplante and Sabatini, 2012). Recent studies have revealed that mTORC1 and mTORC2 share a few functions but exhibit many distinct functions in Purkinje cells (Thomanetz et al., 2013; Angliker et al., 2015). A deficiency in mTORC2 results in multiple CF innervation but does not affect cerebellar LTD. A deficiency in mTORC1 results in a progressive loss of Purkinje cells, while its involvement in cerebellar LTD remains unknown. In other words, the dendritic atrophy of Purkinje cells induced by a deficiency in mTORC1 or mTORC2 is similar to that observed in DGKγ KO mice, although DGKγ KO mice do not exhibit the multiple CF innervation seen in mTORC2 KO mice and the loss of Purkinje cells seen in mTORC1 KO mice. Therefore, it is possible that PA-mTOR signaling also interferes with the phenotype of DGKγ KO mice.

**Figure 9.**
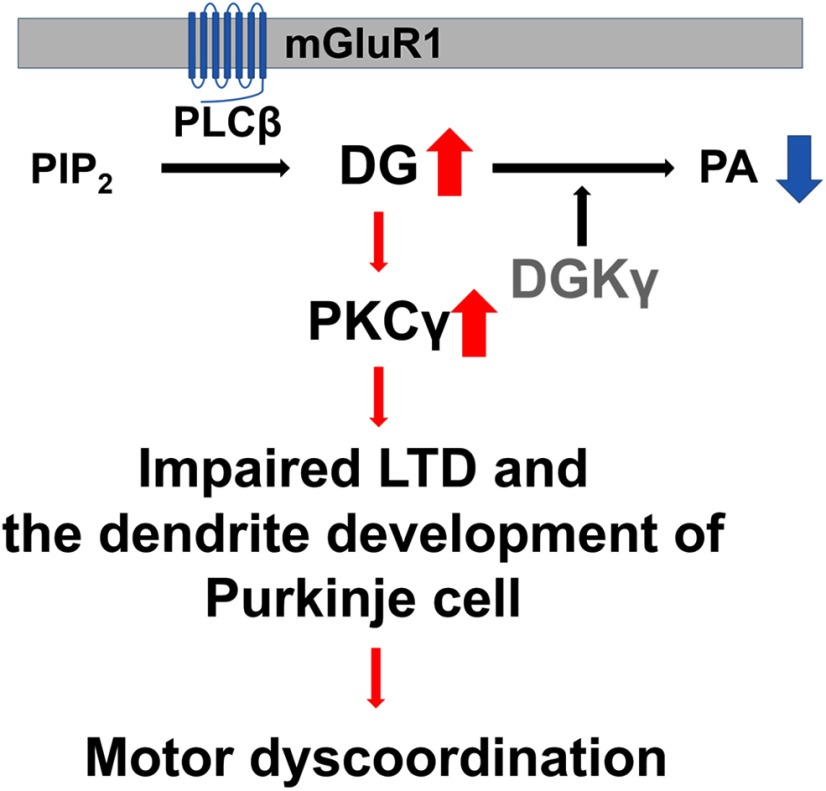
Schematic illustration of motor dyscoordination in DGKγ KO mice. DGKγ deficiency decreases PA levels and increases DG levels. Increased DG constitutively activates PKCγ, resulting in impaired LTD and the dendritic development of Purkinje cells. These alterations are manifested as motor dyscoordination in DGKγ KO mice.

In summary (Fig. 9), the increase in DG levels caused by DGKγ deficiency aberrantly activates PKCγ Activated PKCγ impairs LTD and the dendritic development of Purkinje cells, resulting in motor dyscoordination in DGKγ KO mice. We showed for the first time the importance of DGKγ in cerebellar LTD and the dendritic development of Purkinje cells through the regulation of PKCγ activity and its contribution to cerebellar motor coordination.

## Synthesis

Reviewing Editor: Christian Hansel, University of Chicago Decisions are customarily a result of the Reviewing Editor and the peer reviewers coming together and discussing their recommendations until a consensus is reached. When revisions are invited, a fact-based synthesis statement explaining their decision and outlining what is needed to prepare a revision will be listed below. The following reviewer(s) agreed to reveal their identity: Tomoo Hirano.

The authors reported that DGKγ knockout mice show motor discoordination, impaired synaptic plasticity LTD, and abnormal Purkinje cell morphology. They also suggested that up-regulation of PKCγ activity is related to these phenotypes.

The authors generated brain wide DGKγ KO mice with cerebral and cerebellar DGKγ deficiency. Motor coordination and cerebellar parallel fiber-Purkinje cell synapse LTD were impaired in the KO mice. The number of branches and the total length of the dendrites of the Purkinje cell in the KO mice were lower than those in WT mice. This morphology deficit was also observed in the primary culture of Purkinje cells. Pharmacological activation and inhibition of PKC in the culture condition reduced and mimicked the morphologic deficit in the KO mice. They found an upregulation of PKCγ activity, but not PKCα, in the cerebellum of the KO mice. Motor coordination deficits were not observed in DGKγ expressing mice. Based on these data, the authors claim that the increase in DG caused by DGK deficiency activated PKC and this activated PKC impairs LTD and PC morphology, resulting in motor coordination deficit in the KO mice.  

Major comments: However, the authors did not show any direct data supporting that the activated PKCγ impairs LTD. The pharmacological and molecular intervention of PKCγ in LTD induction should be done to support their idea. They described that the activated PKCγ-mediated inhibition of slow EPSC may underlie the LTD deficit. They may observe that the slow EPSC is also reduced in the KO mice. This problem should be experimentally addressed if possible. Otherwise, it needs to be addressed in a caveat discussion. The reduction of slow EPSC and LTD deficit in the KO mice can be reversed by PKCγ intervention. The authors also should discuss a recent paper of DGK-PKC mediated mechanisms in Purkinje cell LTD. Lee D et al (J Neuroscience, 2015) showed functional and physical interaction of DGK and PKC is required for cerebellar LTD. Authors should discuss the possible involvement of DGK-PKC interaction in the LTD induction.   The authors suggest two deficits, LTD and morphology of Purkinje cell as a cause of motor coordination deficit in the brain wide KO mice. Purkinje cell-specific rescue of LTD and morphology in the KO mice is needed to support the causal relationship. Otherwise, authors should tone down when they are interpreting these correlation data between cellular mechanisms and behavior.   Is the DGKγ expressed selectively in the Purkinje cells? In Fig.1, cerebellar granule cell layer of WT shows positive staining of DGKγ compared to KO.   How can the increase of DG selectively activate PKCγ but not PKCα? Both are known to be activated DG.

Minor comments:

1, On P4 line 66-68, authors wrote “we previously reported that--------(Yamaguchi et al., 2006), which allows reviewers to guess who is an author. This sentence should be rewritten.   2, On P26-27 authors discuss that PKCγ up-regulation might suppress LTD through inactivation of TRP3. Another possibility is that up-regulation of PKCγ might saturate LTD and occlude additional LTD induction. This idea might be discussed.   3, Authors also should discuss how abnormal morphology (retarded dendrites) of Purkinje cells affect motor coordination.   4, Age of animals should be stated in both behavioral and electrophysiological experiments.   5, P12 line 205,206, the timing of PF stimulation during 50 ms depolarization should be written.   6, P19 line 328, “equal to base line levels (---p = 0.0003”. If they were equal, how significant difference could be detected? The sentence should be rewritten.   7, Figure 3, representative EPSC traces should be presented.   8, Figure 1D and Figure 4A, scale bars are too faint.   9, Figure 5A a scale bar is missing.   10, Figure 5 legend line 732, “Con means control” is an explanation for (B).   11, Format of references should be corrected. Use of capital letters is not coherent.   12, There are many grammatical and typographic errors, and use of words is sometimes inappropriate. I recommend thorough English editing. Examples of errors or inappropriate expressions follow.   P1 line 13, 14, “Purkinje cells----were retracted” “the retraction of -Purkinje cells”. Were dendrites of Purkinje cells retracted?   P2 line 20, “DGKγ is involved in---the morphology of Purkinje cells”   P2 line 26 “morphology of Purkinje cells are important for motor function”   P2 line 28 “we are still inadequate to understand”   P8 line 132, “cerebellums”   P10 line 170, “2days 8”. What is “8”?   P14 line 241, “coated with --- solution”.   P14 line 251, “tritonized with 0.3% Triton”   P16 line 279, “eeagent”   P16 line 281,282, “the amount of ----was detected”   P16 line 340, “CF innervation of Purkinje cells is also responsible for motor coordination.   P20 line 347, 348, “These results indicated that DGKγ was not involved in CF innervation in Purkinje cells”. I think that the results showed that DGKγ knockout did not affect pruning processes of multiple CF innervation to a Purkinje cell.   P21 line 369, “the morphological impairments in Purkinje cells”   P23 line 397,398, “PKCγ activation is key for the morphological impairment of primary cultures”   P23 line 402, 403, “DGKγ contributes to the morphology of dendrites”   P23 line 412, “affencted”   P26 line 460, “costimulation from PFs and CFs   P27 line 427, “Go6976 normalized the retraction of dendrites”   P27 line 480, “DGKγ contributes to the morphology of Purkinje cells”   P27 line 484, “the disruption of the transmission of refined motor programs”   P29 line 506, “the sensitivity of rapamycin”   P36 line 684,685, “gene genome”   P38 line 719, “fluorescent-dyed Purkinje cells”   P39 line 743 “phosphorylation to total protein ratios”   P39 line 751, “Cerebral and Cerebellar from WT”   P40 line 762, “alterations manifest as discoordination in DGK KOγ mice"
